# Effects of Kamikihito and Unkei-to on Sleep Behavior of Wild Type and Parkinson Model in *Drosophila*

**DOI:** 10.3389/fpsyt.2017.00132

**Published:** 2017-07-31

**Authors:** Kumpei Ito, Haruhisa Kawasaki, Takahiro Suzuki, Tsubasa Takahara, Norio Ishida

**Affiliations:** ^1^Institute of Chronobiology, Foundation for Advancement of International Science, Tsukuba, Japan; ^2^Graduate School of Life and Environmental Sciences, Tsukuba University, Tsukuba, Japan; ^3^Ishida Group of Clock Gene, Biomedical Research Institute, National Institute of Advanced Science and Technology (AIST) 6 Central, Tsukuba, Japan

**Keywords:** *Drosophila*, Kampo medicine, Parkinson’s disease, sleep disorders, neurodegenerative diseases

## Abstract

Parkinson’s disease (PD) is the second most common neurodegenerative disease, and it is associated with sleep behavior disorders. In *Drosophila melanogaster* disease model, human α-synuclein A30P overexpressing flies (A30P PD model) have been shown for levy body aggregation and movement disorders. We measured sleep rhythms in the A30P PD model flies using the *Drosophila* Activity Monitoring system and found that they develop sleep defects at 20 days after eclosion. Furthermore, the total amount of sleep is significantly reduced in middle-aged PD model flies and the reduction has been attributed to nighttime sleep. The number and length of sleep bouts also decreased in middle-aged A30P PD model flies. Feeding of the oriental traditional herbal medicines (Kampo), Kamikihito and Unkei-to significantly ameliorate the level of sleep defects in A30P PD model flies. The Kamikihito and Unkei-to recovered 60-min sleep bouts number in the A30P PD model flies to the level of young (5 days after eclosion) flies. Kamikihito recovered sleep both in wild-type and PD model flies. Unkei-to ameliorates not only sleep but also motor function in PD model flies. The data suggest that Kamikihito and Unkei-to might be useful for the sleep defects in human PD patients as well as healthy human.

## Introduction

Parkinson’s disease (PD) is a neurodegenerative disease characterized by the loss of dopaminergic neurons in the substantia nigra pars compacta, protein aggregation in neurons (Lewy bodies), and movement disorders, such as tremor at rest, bradykinesia, and rigidity. In addition, PD causes non-motor symptoms such as depression, impaired olfaction, and sleep deficits (1). Over 90% of patients with PD develop sleep rhythm abnormality such as increased daytime sleep, sleep fragmentation, and the loss of slow-wave sleep (SWS) (2). Sleep quality is very important to maintain homeostasis, and sleep deprivation in mice causes increased oxidative stress and changes in antioxidant enzyme activities (3). Furthermore, sleep disorder deficits have been suggested signature for early PD, because patients with REM sleep behavior disorder are associated with a high risk of developing PD (4, 5). These human data suggest that the possibility of early sleep deficits may be occurred in *Drosophila* PD models.

In *Drosophila melanogaster* PD models, short-term memory deficits following sleep deprivation have been reported (6). Furthermore, sleep alteration was reported in overexpression of wild and A53T mutated human α-synuclein (7). However, sleep abnormality was not determined in α-synuclein A30P PD model flies. In modern society, dementia is one of the severe social problems. Up to 80% of PD patients progress to Lewy body dementia (8). Mutated human α-synuclein is known for insoluble protein aggregates in familial PD (9). *Drosophila* models of PD overexpressing human α-synuclein (SNCA)-A30P (A30P PD) or -A53T in whole neurons developed Lewy body aggregation and movement disorders (9).

Japanese traditional herbal medicine (Kampo) originated in traditional Chinese medicine (TCM) that has been established for over 2,000 years. Around 1,500 years ago, TCM was introduced into Japan, where it underwent further development after merging indigenous folk medicine. Kampo medicine is a mixture of many herbs, and their various drug components may act coordinately. Kampo is now established and reproduced by pharmaceutical companies under quality-controlled conditions. Thus, we screened the ability of various types of Kampo to ameliorate sleep behavior abnormality in PD model flies.

Here, we showed early sleep deficits in *Drosophila* PD models that overexpress human mutated α-synuclein (A30P PD model). We then screened several substances derived from Kampo and bio-modulators to identify those that could ameliorate sleep deficits in A30P PD model flies.

## Materials and Methods

### Fly Rearing and Crosses

*Drosophila* strains were maintained as described previously (10). Flies were reared in vials of standard yeast cornmeal at 25°C and entrained to LD 12. All experiments proceeded on mated male flies. Transgenic females carrying *UAS-SNCA-A30P* or *UAS-SNCA-WT* constructs were crossed with males carrying the pan-neuronal driver *elav-GAL4* to generate PD model flies expressing human mutated α-synuclein protein. We used human α-synuclein A30P overexpressing PD model *Drosophila* (9), which showed adult-onset loss of dopaminergic neurons, locomotor dysfunction, and filamentous inclusions bodies containing α-synuclein in brain. The controls were *w; elav-Gal4/*+, *SNCA A30P/*+, or *UAS-SNCA-WT/CyO* flies. Canton-S (WT) flies were used as a control in drug screening. *Drosophila* strains were obtained from Bloomington *Drosophila* Stock Center.^1^

### Kampo Medicines and Chemicals

Saiko-ka-ryukotsu-borei-to (Saiko) and Kamikihito are types of medicine that are used to treat climacteric symptoms and depression, respectively (11–14), whereas both Unkei-to and AC-Negia are used to treat menstrual irregularity (15, 16) (Table 1). Saiko-karyuukotsu-borei-to (JAN code. 4987241304295), Kamikihito (JAN code. 4987241304288), Unkei-to (JAN code. 4987241304615), and AC-Negia (JAN code. 4987241112562) were provided from Masao Hashimoto (ROHTO Pharmaceutical Co., Ltd.). These products are marketed as tablets containing mixture of natural products chemistry according to traditional Kampo prescription. Myo-inositol and d-pinitol are components of ice plants (*Mesembryanthemum crystallinum*), which affect the circadian rhythm of mating behavior in *Drosophila* and mammalian cells (10). These two compounds were purchased from Wako Pure Chemical Industries, Ltd. The product codes are 094-00281 and 320-75401, respectively. l-alpha-glycerophosphatidylcholine (α-GPC) and phosphatidylcholine docosahexaenoic acid (PC-DHA) are extracts of salmon egg membranes (provided by NOF Corporation).

**Table 1:** Summary of Kampo medicines used for the experiments.

Name of used medicine	Usage
Kamikihito	Depression, insomnia, neurosis, loss of appetite, amnesia
Unkei-to	Menstrual irregularity, sterility, climacteric symptoms
Saiko (Saiko-ka-ryukotsu-borei-to)	Neuropsychiatric disorders, erectile dysfunction, malignant hypertension, climacteric symptoms
AC-Negia (Keishi-bukuryo-ganryo-kayoku-inin)	Skin pigmentation, menstrual irregularity
l-alpha-glycerophosphatidylcholine (α-GPC)	Alzheimer-type dementia
PC-DHA	Sleep improvement

*Substances were selected based on psychiatric effect. α-GPC and PC-DHA are not belonging to Kampo*.

### Fly Food Preparation

Fly food was prepared as described previously (10). Boiled standard medium consisting of 8% corn meal, 5% glucose, 5% dry yeast extract, 0.64% agar was supplemented with 0.5% propionic acid and 0.5% butyl p-hydroxybenzoate. Kampo tablets were powdered with mortar and pestle. Each Kampo and substance were dissolved in distilled water and added to standard medium at final concentrations as described in figure legend (Kamikihito, Unkei-to, Saiko, and AC-Negia are 1.6 × 10^−3^ g/mL; myo-inositol, d-pinitol, α-GPC, and PC-DHA are 2.0 × 10^−4^ g/mL). Flies were fed the media containing these substances after eclosion until experiment. Substances were selected based on psychiatric effect (see Table 1). α-GPC and phosphatidylcholine docosahexaenoic acid (PC-DHA) are used for therapy of Alzheimer-type dementia and sleep improvement, respectively.

### Assays of Sleep Behavior

Sleep behavior was recorded as described previously (17). Male flies with different genotypes were placed in the *Drosophila* Activity Monitoring (DAM) system (TriKinetics, Waltham, MA, USA) for 3 days (*n* ≥ 24). Locomotor activity was measured in 1-min bins, and sleep was traditionally defined as ≥5 min of consolidated inactivity (18) and ≥60 min of such inactivity for more detailed analyses. Sleep analysis software provided by M. Shimoda (National Institute of Agrobiological Science) was used to analyze the *Drosophila* locomotor activity data and sleep data. All experiments were tested by using male flies.

### Sleep Deprivation and Climbing Assay

Flies were fed with Kampo medicine soon after eclosion. Sleep deprivation experiments were repeated in 1-min interval rotation (350 rpm) by using rotating shaker (CUTE MIXTURE CM-1000; EYELA Co., Ltd., Tokyo, Japan) between ZT 12 and 16 to PD model flies from day 20 to day 42 after eclosion. This method was modified from Shimizu et al. (19). Climbing activity was determined as described (9) at day 45 after eclosion.

### Statistical Methods

**Figure 1:**
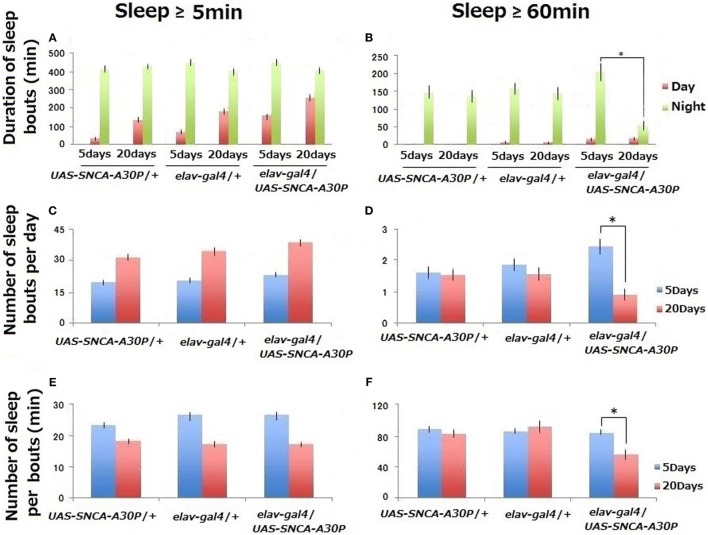
Sleep behaviors of controls (*UAS-SNCA-A30P/*+ and *elav-gal4/*+) and A30P Parkinson’s disease model (*elav-gal4/SNCA-A30P*) *Drosophila* males during 3 days. Comparison was done from 3 to 5 days and from 18 to 20 days after eclosion (*n* ≥ 24). Sleep amount/day **(A,B)**, number **(C,D)**, and duration **(E,F)** of sleep bouts. Statistical data by Student’s *t*-test are expressed as mean ± SEM. We have repeated this experiment for five times. * represents significant differences (*p* < 0.05).

**Figure 2:**
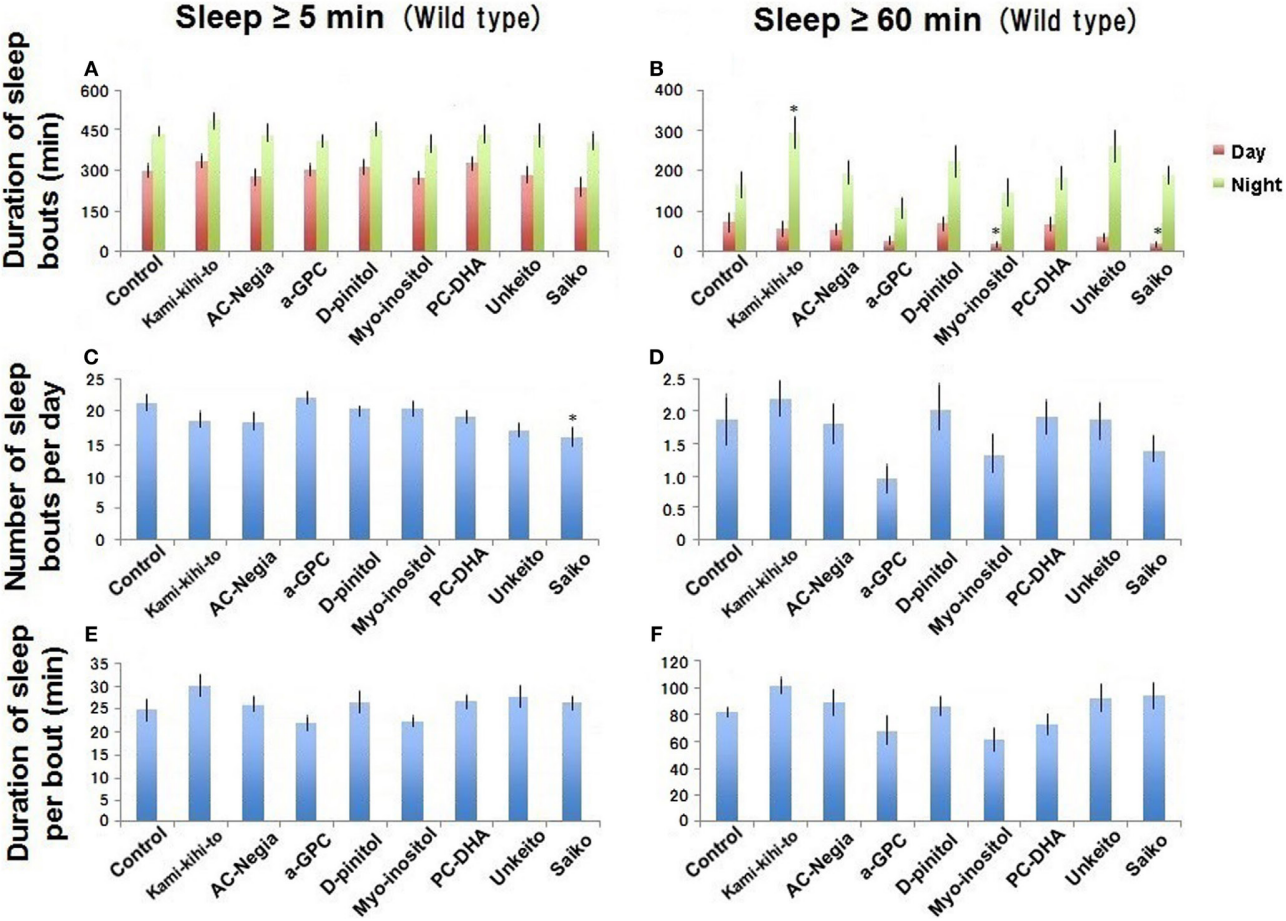
Sleep behavior in wild-type (Canton-S) male flies was assessed between 3 and 5 days after eclosion (*n* = 15 flies as control). Canton-S flies fed with 1.6 × 10^−3^ g/mL of Kamikihito (*n* = 16 flies), Unkei-to (*n* = 16), Saiko (*n* = 15), and AC-Negia (*n* = 15), as well as 2.0 × 10^−4^ g/mL of myo-inositol (*n* = 15), d-pinitol (*n* = 15), l-alpha-glycerophosphatidylcholine (α-GPC) (*n* = 16), and PC-DHA (*n* = 16) in standard cornmeal yeast medium after eclosion. Sleep amount/day **(A,B)**, number **(C,D)**, and duration **(E,F)** of sleep bouts. Results are expressed as mean ± SEM. Data were statistically analyzed by one-way ANOVA, followed by Dunnett’s *post hoc* test. Obtained *F* values are as follows: **(A)** [day: *F*(8,130) = 1.13; *p* = 0.34, night: *F*(8,130) = 0.69; *p* = 0.69], **(B)** [day: *F*(8,130) = 2.18; *p* = 0.03, night: *F*(8,130) = 2.95; *p* = 0.0045], **(C)** [*F*(8,130) = 2.60; *p* = 0.011], **(D)** [*F*(8,130) = 1.77; *p* = 0.088], **(E)** [*F*(8,130) = 1.63; *p* = 0.11], **(F)** [*F*(8,130) = 2.29; *p* = 0.024]. * represents significant differences (*p* < 0.05).

**Figure 3:**
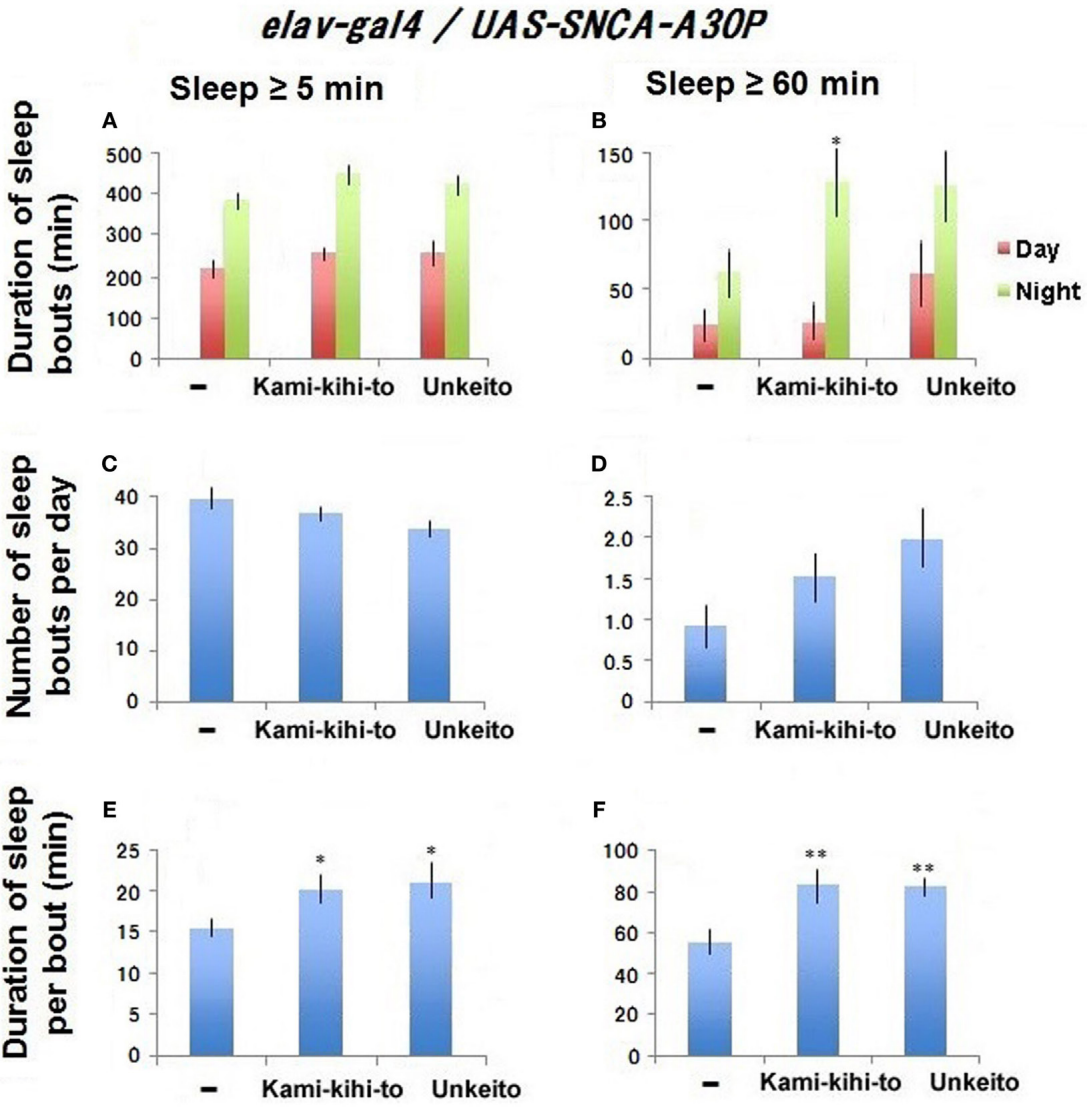
Sleep behaviors in A30P Parkinson’s disease (PD) model male flies fed with 1.6 × 10^−3^ g/mL Kamikihito (*n* = 30 flies) and Unkei-to (*n* = 26) in medium during 10–20 days after eclosion. A30P PD model flies with standard food are used as control (*n* = 31). Sleep behaviors were measured between 18 and 20 days after eclosion. Sleep amount/day **(A,B)**, number **(C,D)**, and duration **(E,F)** of sleep bouts. Results are expressed as mean ± SEM. Data were statistically analyzed by one-way ANOVA, followed by Dunnett’s *post hoc* test. Obtained *F* values are as follows: **(A)** [day: *F*(3,115) = 0.91; *p* = 0.43, night: *F*(3,115) = 1.78; *p* = 0.15], **(B)** [day: *F*(3,115) = 1.26; *p* = 0.28, night: *F*(3,115) = 3.65; *p* = 0.014], **(C)** [*F*(3,115) = 3.34; *p* = 0.021], **(D)** [*F*(3,115) = 1.65; *p* = 0.18], **(E)** [*F*(3,115) = 3.42; *p* = 0.019], **(F)** [*F*(3,115) = 5.18; *p* = 0.0021]. * and ** represent significant differences (*p* < 0.05 and *p* < 0.01, respectively).

**Figure 4:**
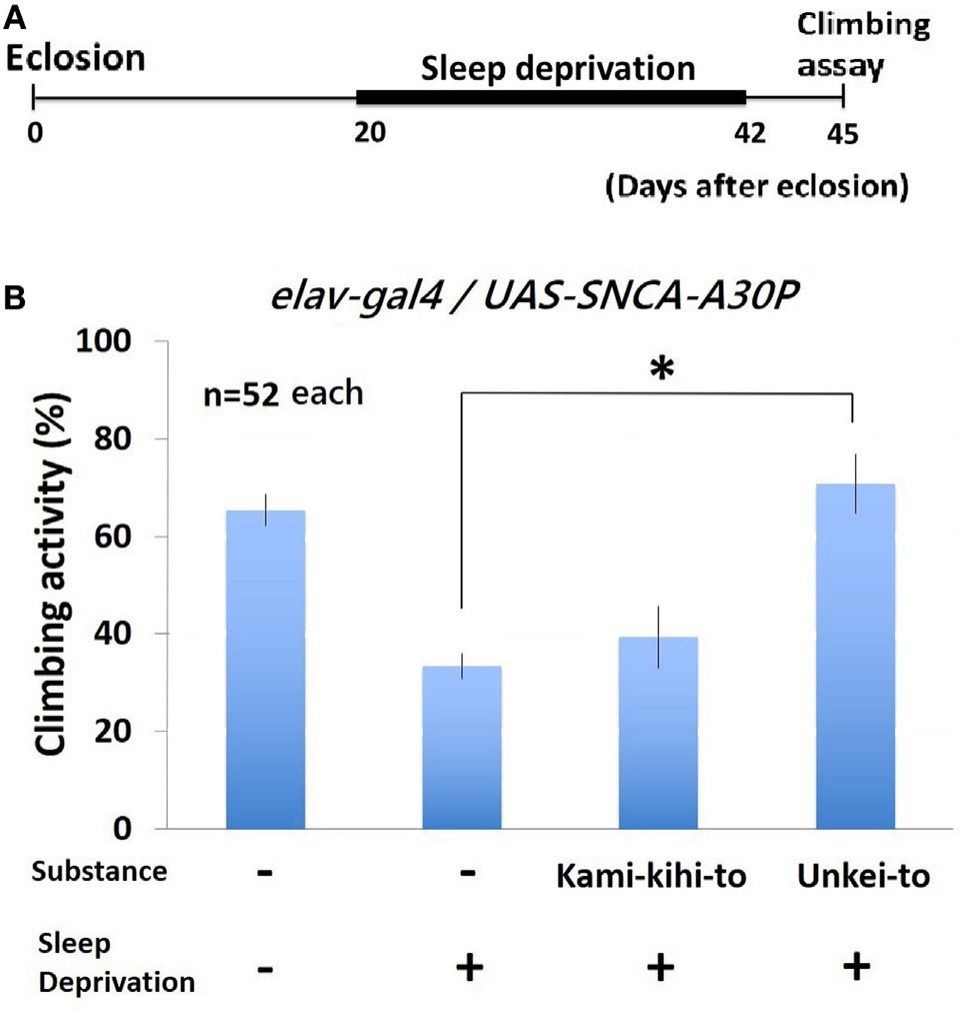
Climbing activity of A30P Parkinson’s disease (PD) model flies was dramatically decreased by sleep deprivation. **(A)** Schematics of sleep deprivation assay experiment. A30P PD model flies were given substances (0.25%) since eclosion. From 20 to 42 days after eclosion, sleep deprivation was repeated minutely between ZT 12 and 16. Climbing assay was performed at 45 days after eclosion. **(B)** Climbing activity of A30P PD model *Drosophil*a was recovered by Kampo medicine. Each experiment was repeated for three times (*n* = 52 flies each). * indicates significant differences (*p* < 0.05).

Statistical analysis was performed using Excel 2010 (Microsoft, Seattle, WA, USA) with the add-in software Statcel 3 (Yanai H. Statcel, Available: The useful add-in software^2^ forms on Excel. 3rd ed. Tokyo, Japan: OMS; 2011. pp. 172–175). Results are expressed as mean ± SEM. Data were statistically analyzed by Student’s *t*-test (Figures 1 and 4; Figures S1 and S2 in Supplementary Material) or one-way ANOVA, followed by Dunnett’s *post hoc* test (Figures 2 and 3). Obtained *F* values are shown in the Figure legends. For all tests, a *p* < 0.05 was considered statistically significant.

## Results

### Sleep Abnormality in A30P PD Model Fly

The GAL4-UAS system is widely used in *Drosophila* to express gene under control of tissue-specific promotor. Neuron-specific promoter, *elav*, drives human mutant α-synuclein A30P in this experiment.

*Drosophila* Activity Monitoring system data indicate that the total amount of sleep in ≥5-min bouts was higher in middle-aged (20 days after eclosion) than in young (5 days after eclosion) control flies (*UAS-SNCA-A30P/*+ and *elav-gal4/*+) (Figure 1A). This increase was mainly due to daytime sleep (shown as red).

The similar increase was evident in A30P PD model flies (*elav-gal4/SNCA-A30P*) at 20 days, but the daytime sleep level at 5 days was similar to that of control flies at 20 days (Figure 1A). In contrast, the total amount of 60-min bouts of sleep was significantly reduced in middle-aged (20 days) A30P PD model flies (Figure 1B). This decrease was mainly due to nighttime sleep indicating that sleep behavior disorders were detected even in A30P PD model flies.

The number of sleep bouts increased, and the length of sleep per bouts in ≥5-min decreased with aging in controls (Figures 1C,E). These data were consistent with other findings of WT flies (20, 21). In middle-aged (20 days) A30P PD model flies, the number and length of ≥60-min sleep bouts decreased significantly (Figures 1D,F). Thus, sleep became fragmented in middle-aged A30P PD model flies.

### Screening for Sleep-Promoting Substances

To reduce sleep deficit in A30P PD model flies, we screened several candidates of the sleep promoters. None of the test materials affected the amount and length of ≥5-min sleep bouts in WT flies (Canton-S) at 5 days after eclosion (Figures 2A,E). However, Saiko reduced the number of ≥5-min sleep bouts (Figure 2C). The data indicate that these types of Kampo might improve sleep rhythm abnormality. Kamikihito increases the amount of long (≥60 min) sleep bouts during the nighttime in WT flies (Figure 2B). Myo-inositol and Saiko reduced the amount of ≥60-min bouts of daytime sleep in WT flies (Figure 2B), indicating that they might play roles in wakefulness during day. Saiko has been used to treat insomnia previously (22), perhaps *via* its ability to promote daytime wakefulness.

### Kamikihito and Unkei-to Rescued Bouts of Long Sleep in Aged A30P PD Model Flies

To see the effect of sleep-promoting substances to A30P PD model flies, *Drosophila* models were fed with these substances for 10–20 days after eclosion, and then sleep behavior was measured between days 18 and 20 by using DAM system. Kamikihito significantly recovered nighttime sleep in middle-aged A30P PD model flies (Figure 3B). Kamikihito and Unkei-to recovered ≥60-min sleep bout length in middle-aged model A30P PD flies to the level of that in young flies at 5 days after eclosion (Figures 1F and 3F). The data indicate that Kamikihito and Unkei-to might be useful for ameliorating sleep abnormality in A30P PD model. However, Kamikihito is effective to recover quality of sleep in wild-type fly.

### Climbing Activity of A30P PD Flies after Sleep Deprivation Recovered by Administration of Unkei-to

To determine whether the motor dysfunction in PD model flies will be recovered by Kampo medicines, we measured climbing activity after sleep deprivation. Climbing activity of A30P PD flies were assayed at 3 days after sleep deprivation for 22 days (Figure 4A). Sleep deprivation decreased climbing activity of wild-type (Figure S1 in Supplementary Material) and A30P PD model *Drosophila* seriously (Figure 4B). The decreased climbing activity of A30P PD flies significantly recovered by feeding Unkei-to (Figure 4B). Kamikihito also increased the climbing activity of A30P PD flies, but this increase is not significant.

## Discussion

### Sleep Defects in A30P PD Model Flies

As described in Section “Materials and Methods,” human α-synuclein A30P overexpressing PD model *Drosophila* showed the essential features of human disorder (9, 18). In this paper, we showed sleep abnormality in middle-aged *Drosophila* models of A30P PD by using DAM system. Two sleep stages have recently been reported in *Drosophila* (23, 24). A30P PD model flies at 20 days after eclosion decreased long sleep significantly during the nighttime. These findings suggest that the stage of long bouts of sleep was adversely affected in *Drosophila* models of A30P PD. This long sleep is very similar to SWS in mammals (23, 24). As PD patients have been reported for decreased SWS (2), the decrease of long sleep in A30P PD flies suggests the phenotype in PD between *Drosophila* and mammals might be similar (2, 23, 24).

### Myo-Inositol and Saiko Affect Circadian Behavior

We showed that myo-inositol and Saiko reduced daytime sleep in WT flies. As many neurodegenerative and psychiatric disorders disrupt sleep–wake cycles (25, 26), these two medicines might help to maintain the amplitude intensity of sleep–wake cycles in patients with the disorders. Saiko is used to treat insomnia previously (22), because the ability of Saiko promotes daytime wakefulness in humans. Such promotion effect in daytime might contribute to enhance the nighttime sleepiness in *Drosophila* (Figure 2). However, we still do not know the precise mechanism for these drugs to A30P PD model flies.

### Kamikihito and Unkei-to Increased Sleep Quality in A30P PD Model Flies

Here, we showed that Kamikihito and Unkei-to rescued sleep quality in middle-aged *Drosophila* models of A30P PD, suggesting that these Kampos may be useful for recovering the sleep quality of PD patients in humans. However, we need further clinical study in humans, because the molecular mechanisms of sleep between two species are not the same completely.

The common ingredients of two Kampos were licorice, ginger, angelica root, and ginseng root. The data suggest that sleep-promoting substance might be included in these ingredients. But, contents of these materials are different in Kamikihito and Unkei-to, suggesting that it is very difficult to determine a single substance for sleep-promoting effect.

Unkei-to relieves stress by suppressing CRF (corticotropin-releasing factor) (27), so that Unkei-to may induce sleep by suppressing stress. In addition, it has been reported that Kamikihito improves insomnia (28). Considering with the above papers, our data suggest that the Kampos (Kamikihito and Unkei-to) may be useful for the sleep abnormality in A30P PD model flies as well as PD patients.

The decreased climbing activity of A30P PD flies after sleep deprivation recovered by feeding Unkei-to (Figure 4B). The data also suggest Unkei-to might be useful in improving motor function as well as sleep quality. As we did not check the effect of these Kampos on α-synuclein aggregation in this study, we did not know the molecular mechanism why these Kampos improve sleep defects in fly. This study will bring a new insight into a role of Kampo, Kamikihito, and Unkei-to in sleep defects in PD models and wild-type fly.

## Author Contributions

NI, HK, KI, and TS designed the study. NI, KI, and HK performed paper writing. KI, HK, and TT carried out experiments. NI, KI, TS, and HK performed data analysis.

## Conflict of Interest Statement

The authors declare that the research was conducted in the absence of any commercial or financial relationships that could be construed as a potential conflict of interest.

## Footnotes

^1^http://fly.bio.indiana.edu/.

^2^http://www.oms-publ.co.jp/.

## Supplementary Material

Figure S1 Climbing activity of wild-type flies was reduced by sleep deprivation. Climbing assay was performed as shown in Figure 4A. Climbing activity of wild type (Canton-S) was also decreased by sleep deprivation in Figure 4B (*n* = 18 flies each). * indicates significant differences (*p* < 0.05).

Figure S2 Sleep behaviors (sleep amount/day) of flies expressing human wild-type α-synuclein on neuron (*UAS-SNCA-WT/elav-gal4*: *n* = 29 flies each) and control flies (*UAS-SNCA-WT/CyO*: *n* = 30 flies each) during 3 days. Comparison was done from 3 to 5 days and from 18 to 20 days after eclosion. Statistical data are expressed as mean ± SEM. * and ** represent significant differences (*p* < 0.05 and *p* < 0.01, respectively).
