# Supplementary figures and images for: Effects of Kamikihito and Unkei-to on Sleep Behavior of Wild Type and Parkinson Model in Drosophila
Source: Front Psychiatry. 2017 Jul 31;8:132. doi: 10.3389/fpsyt.2017.00132 (PMC5534454; doi:10.3389/fpsyt.2017.00132)

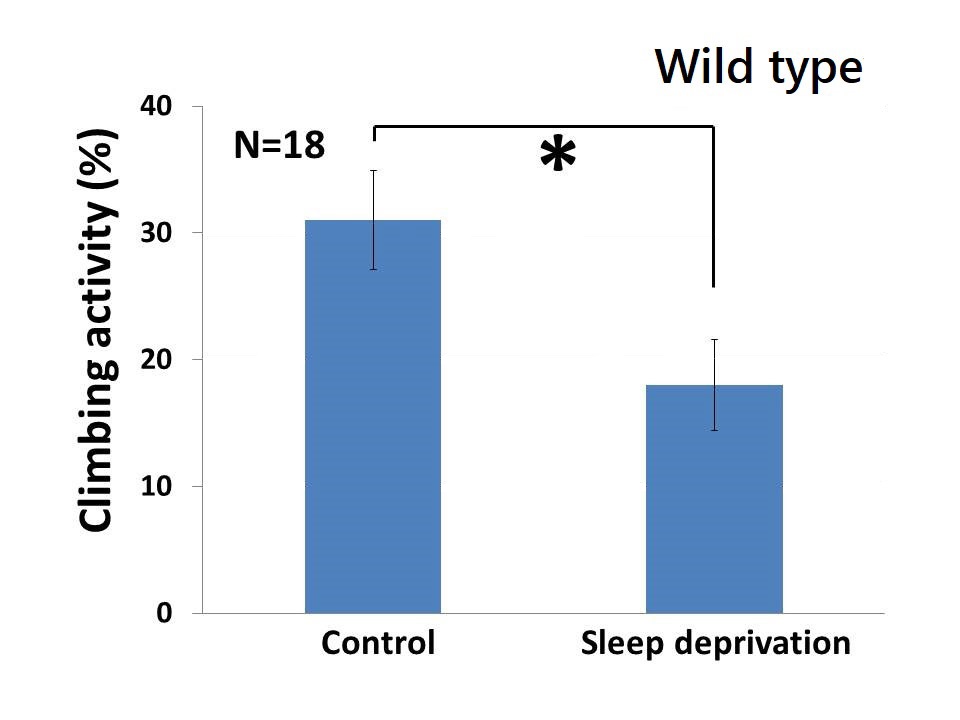

Supplement: Figure S1 — Climbing activity of wild-type flies was reduced by sleep deprivation. Climbing assay was performed as shown in Figure 4A. Climbing activity of wild type (Canton-S) was also decreased by sleep deprivation in Figure 4B (n = 18 flies each). * indicates significant differences (p < 0.05). [file image_1.jpeg]

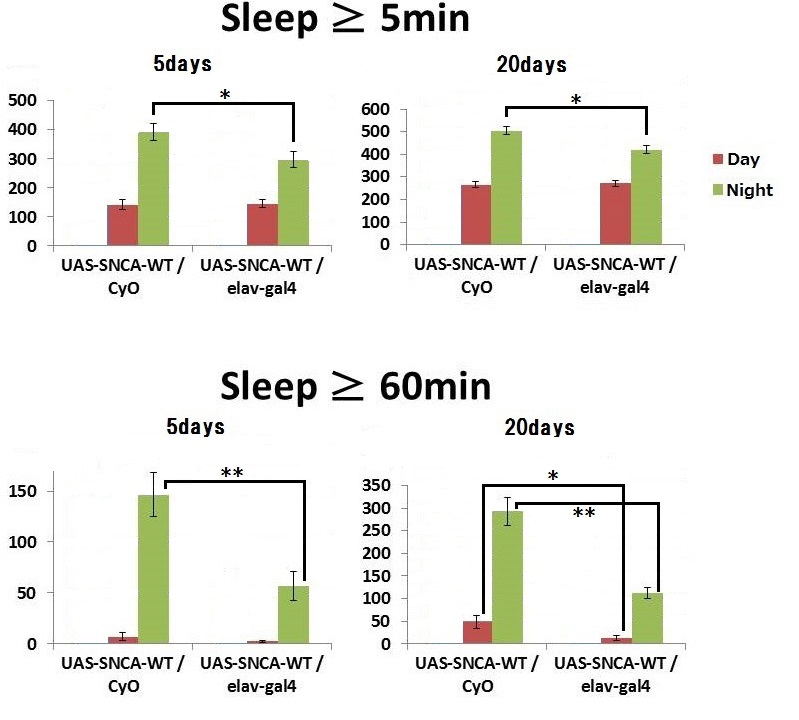

Supplement: Figure S2 — Sleep behaviors (sleep amount/day) of flies expressing human wild-type α-synuclein on neuron (UAS-SNCA-WT/elav-gal4: n = 29 flies each) and control flies (UAS-SNCA-WT/CyO: n = 30 flies each) during 3 days. Comparison was done from 3 to 5 days and from 18 to 20 days after eclosion. Statistical data are expressed as mean ± SEM. * and ** represent significant differences (p < 0.05 and p < 0.01, respectively). [file image_2.jpeg]
